# BIOSOCIAL PATHWAYS AFFECTING HEALTH AND AGING IN THE HEALTH AND RETIREMENT STUDY

**DOI:** 10.1093/geroni/igad104.0718

**Published:** 2023-12-21

**Authors:** Eric Klopack, Helen Meier

## Abstract

Representative population studies, like the Health and Retirement Study (HRS), are gathering large amounts of biomarker and multi-omic data. This presents a unique opportunity to investigate how population-level aging effects is driven by social inequality. This research has the capacity to uncover targets for intervention that could result in extending longevity and healthy life span and reducing inequalities in aging. There has been substantial research and progress in identifying biological processes underlying age-related decline, morbidity, and mortality (the so-called biological hallmarks of aging); however, despite this progress, demographic, social, and psychological factors (the so-called social hallmarks of aging) are substantially associated with cognitive aging and ADRD above-and-beyond genetic and biological factors There is, therefore, a need for research that carefully integrates the social and biological hallmarks of the human aging processes. This symposium highlights research that investigates interactions between biological and social processes that lead to inequalities in health and aging among older adults utilizing recently released data from the HRS Venous Blood Study (a nationally representative sample of US adults over age 55). Included are papers investigating the role of timing of childhood adversity in affecting adult DNA methylation patterns, how childhood parental loss is associated with accelerated epigenetic aging, how socioeconomic status patterns expression of genes related to inflammation and cellular senescence, and how sets of age-related biomarkers can explain sociodemographic inequalities in mortality. These studies contribute to an emerging literature integrating social and biological sciences to better understand inequalities in the aging process.

